# Behavior in Australian Shepherd Dogs Assessed Using the C-BARQ: A Preliminary Study of Associations with Coat Color, Sex, and Neutering Status

**DOI:** 10.3390/vetsci13030299

**Published:** 2026-03-21

**Authors:** Valentina Gazzano, Sofia Maria Alessi, Chiara Santoni, Maria Claudia Curadi, Francesca Cecchi, Stefano Cavallo, Luigi Sacchettino, Danila D’Angelo, Francesco Napolitano, Elisabetta Giannessi, Angelo Gazzano

**Affiliations:** 1 Department of Veterinary Sciences, University of Pisa, 56124 Pisa, Italy; valentina.gazzano@unipi.it (V.G.); s.alessi6@studenti.unipi.it (S.M.A.); maria.claudia.curadi@unipi.it (M.C.C.); francesca.cecchi@unipi.it (F.C.); ste.cavallo@gmail.com (S.C.); elisabetta.giannessi@unipi.it (E.G.); angelo.gazzano@unipi.it (A.G.); 2 Centro Cinofilo 4 Zampe & Co., Loc. Castel Rainero, 1, 10060 Pancalieri, Italy; chiara.santoni@centrocinofilo4zampe.com; 3 Department of Veterinary Medicine and Animal Production, University of Naples Federico II, 80137 Naples, Italyfrancesco.napolitano3@unina.it (F.N.); 4 CEINGE—Biotecnologie Avanzate Franco Salvatore, 80131 Naples, Italy

**Keywords:** C-BARQ, canine behavior, coat color, Australian Shepherd, neutering status

## Abstract

Domestic dogs show substantial behavioral variability shaped by genetic, biological, and environmental factors. Although breed-specific behavioral profiles are documented, associations between coat color and behavior within individual breeds remain poorly understood. This preliminary study characterized the behavioral profile of Australian Shepherd dogs and exploratorily investigated associations between behavior, coat color, sex, and neuter status using the validated Italian C-BARQ. A total of 215 Australian Shepherds aged 1–8.5 years were included; behavioral responses were analyzed using ordinal logistic regression with sex, neuter status, and coat color as predictors. Overall, Australian Shepherds showed high trainability and low aggression and fear toward humans. Sex emerged as the most consistent predictor: females showed lower odds of high-intensity responses in attachment- and excitability-related behaviors (OR ~0.44–0.56), with context-dependent differences in dog-directed and environmental responses. Neuter status was selectively associated with increased fear-related reactions to noise and stranger approaches (OR ~1.9–2.2) and reduced restlessness during separation (OR ~0.45). Coat color was not associated with generalized behavioral tendencies; item-level exploratory analyses revealed only a limited number of suggestive, context-specific associations in merle-coated dogs, and most analyses did not yield significant effects. All coat-color findings should be regarded as preliminary and hypothesis-generating. These results underscore the context-dependent nature of behavioral variation in Australian Shepherds and highlight the need for confirmatory studies with larger, coat-color-stratified samples.

## Simple Summary

Dogs of the same breed can vary widely in behavior, and such variation may be influenced by biological factors including sex, reproductive status, and coat color. This study investigated everyday behavior in Australian Shepherds using a standardized owner questionnaire. Data were collected from 215 dogs and covered common situations such as interactions with people and other dogs, responses to noises and novel environments, and behavior when left alone. Overall, the breed was described as highly trainable and showed low levels of aggression toward people. Clear differences emerged between males and females, particularly in behaviors related to excitement, attachment to owners, and social engagement. Reproductive status was also relevant: neutered dogs displayed some differences in fear-related behaviors, most notably stronger reactions to loud noises. Coat color was not associated with general temperament or overall behavioral profiles. Exploratory analyses identified a limited number of context-specific associations in merle-coated dogs, particularly in response to unfamiliar situations and during veterinary examinations, although the biological significance of these findings remains unclear. These results suggest that biological factors such as sex and neuter status show more consistent associations with behavior than coat color and highlight the need for confirmatory studies with larger samples.

## 1. Introduction

Domestic dogs (*Canis* *familiaris*) are the result of intense artificial selection, primarily driven by human preferences for specific behavioral traits. As highlighted by Serpell and Duffy, dogs differ from other domestic species in that their evolution has been shaped predominantly by selection for behavior rather than morphology alone [1]. Consistent breed-specific behavioral profiles have been documented across a wide range of studies, from early experimental work by Scott and Fuller [2] to more recent expert- and owner-based surveys [3,4].

Although purebred dogs generally exhibit reduced genetic heterogeneity compared to mixed-breed populations [5,6], the formal establishment of modern breeds occurred relatively recently, mainly during the nineteenth century, alongside the development of kennel clubs and standardized breeding practices [7]. Within this framework, behavioral assessment tools based on owner reports have proven particularly useful for large-scale investigations, as they allow the collection of standardized data across diverse everyday contexts while minimizing the influence of cultural stereotypes [1,3,4,8,9].

Among the available instruments [9,10], the Canine Behavioral Assessment and Research Questionnaire (C-BARQ) is one of the most widely used and validated tools for assessing canine behavior [11]. The questionnaire is based on the assumption that owners, as daily observers of their dogs, can reliably report typical behavioral responses across a range of social and non-social situations [12]. The C-BARQ has recently been translated and validated in Italian [13].

Beyond breed-specific behavior, increasing attention has been devoted to the possible association between coat color and behavioral traits. Variation in pigmentation is a well-recognized component of the so-called “domestication syndrome”, a set of correlated morphological, physiological, and behavioral traits observed across domesticated species during the evolutionary process of domestication [14,15,16]. The neural crest hypothesis proposes that selection for tameness affected the development of neural crest cells, leading to pleiotropic effects on pigmentation, stress reactivity, sensory systems, and behavior [16]. Although this framework was developed to explain large-scale evolutionary changes across species rather than variation within a domestic breed, it has motivated broader interest in the relationship between pigmentation-related genes and behavioral traits.

In dogs, coat color is determined by the interaction of multiple genes involved in melanocyte development and function [17], some of which have also been linked to neurological, sensory, and behavioral traits [18]. Whether such genetic links contribute to behavioral differences within individual breeds remains an open question that the present study exploratorily addresses.

Associations between coat color and behavior have been reported in several species, including fish [19], poultry [20], horses [21], cats [22,23], and dogs [24,25,26,27,28].

In dogs, previous studies have described relationships between pigmentation and fearfulness, aggression, and trainability in breeds such as Labrador Retrievers, Cocker Spaniels, and Jindo dogs [26,27,28]. However, findings have often been inconsistent. A growing body of evidence indicates that although coat color genes may show pleiotropic links to neurological and health traits [17,29], direct associations between observable coat color and behavioral characteristics tend to be weak or inconsistent [27,30]. Recent studies suggest that co-selection on pigmentation and behavioral loci during breed formation [5,29,31], together with the highly polygenic architecture underlying personality and hyper-sociability [30,32,33], may largely decouple visible color variation from predictable behavioral differences in individual dogs [27,33]. Moreover, the combined effects of sex and reproductive status have rarely been evaluated within single breeds; therefore, evidence at the within-breed level remains limited.

The Australian Shepherd is a herding breed developed in the United States during the late nineteenth and early twentieth centuries and officially recognized by major kennel clubs only in recent decades [34,35,36]. According to the FCI Standard No. 342, the breed is recognized in four base coat colors—black, red, blue merle, and red merle—each of which may occur with or without white and/or tan markings, resulting in a range of coat pattern variations within each color category [34,35,36]. For the purposes of the present study, dogs were classified according to their base coat color (black, red, blue merle, or red merle), regardless of the presence of white or tan markings. The considerable variation in coat color patterns makes the Australian Shepherd a particularly suitable model for investigating potential associations between coat color and behavior within a single breed.

Despite the breed’s increasing popularity as companion animal, behavioral research specifically focusing on Australian Shepherds remains scarce, and no previous study has systematically evaluated the combined effects of coat color, sex, and neuter status on behavior within this breed.

The aim of the present study was therefore to characterize the behavioral profile of Australian Shepherd dogs and to investigate potential associations between behavioral traits and coat color, while controlling for sex and neutering status, using the validated C-BARQ questionnaire.

Given the observational design and the number of analyses performed, these investigations are intended as exploratory and hypothesis-generating rather than confirmatory. Based on the neural crest hypothesis and previous literature on pigmentation-related behavioral traits within dog breeds, we hypothesized that merle coat color, given its association with pigmentation-related genes and potential sensory effects, would be associated with differences in fear- and arousal-related behaviors compared with non-merle coat colors. We further hypothesized that males and intact dogs would show higher excitability and attachment-seeking behaviors compared with females and neutered dogs, respectively, and that neutered dogs would differ from intact dogs in fear- and arousal-related domains.

## 2. Materials and Methods

### 2.1. The Canine Behavioral Assessment & Research Questionnaire

For this study, the validated Italian version of the C-BARQ was used [13].

The questionnaire was administered online and distributed through social media platforms, breed club pages, and direct sharing among Australian Shepherd breeders and owners. All participants were informed about the general purpose of the study and were guaranteed anonymity. The questionnaire was administered anonymously via an online platform; no personally identifiable information was collected. As participation was voluntary and fully anonymous, formal informed consent was not required. The estimated time required to complete the questionnaire was approximately 10 min.

All questionnaire items required a mandatory response before submission. Dogs outside the age range of 1–8.5 years were excluded prior to analysis, in order to minimize age-related developmental and geriatric effects on behavioral responses. Consequently, no missing data was present in the final dataset and no imputation procedures were necessary.

In addition to the standard C-BARQ items, a general section was included to collect demographic information on each dog, including name, breed, date of birth, sex, coat color (black, blue merle, red, red merle), date of adoption, and the presence of other dogs and/or other animal species in the household. Owners who owned more than one Australian Shepherd were asked to complete the questionnaire for only one dog to avoid potential clustering effects.

The original 100 C-BARQ items were grouped into 15 behavioral subscales, as described by Serpell and Duffy [1]. Depending on the type of behavior assessed, the questionnaire employs two different response formats. Items included in subscales 1, 4, 6, and 7 evaluate the frequency of specific behaviors using a six-point ordinal scale ranging from “not applicable/not observed” to “always”, with scores from 0 to 5 (0 = not applicable, 1 = never, 2 = rarely, 3 = sometimes, 4 = often, 5 = always). This scale provides an estimate of how consistently a behavior is expressed over time.

In contrast, items included in subscales 2, 3, and 5 assess the intensity or severity of behavioral responses, independently of their frequency. These items are rated on a five-point scale ranging from 0 to 4 (0 = none, 1 = mild, 2 = moderate, 3 = intense, 4 = severe), allowing for the evaluation of clinically relevant behaviors even when they occur infrequently.

For all items and subscales, higher scores indicate more problematic behaviors, with the exception of certain items within the trainability subscale, for which higher scores reflect more desirable behavioral traits.

### 2.2. Analysis

Item-level analyses were conducted to capture context-specific behavioral responses that may not be fully represented by aggregated subscale scores.

Although C-BARQ items are ordinal variables, mean values are also reported for descriptive purposes to facilitate comparison with previously published C-BARQ-based studies, in line with established practice in the literature [1,11].

Inferential analyses were conducted using ordinal logistic regression models, which appropriately account for the ordinal nature of the data. Behavioral data derived from individual C-BARQ items were analyzed as ordinal variables reflecting increasing frequency or intensity of the reported behaviors. For each item, a proportional odds model was fitted with behavioral score as the dependent variable and sex (male/female), neuter status (intact/neutered), and coat color (Black, Blue Merle, Red, Red Merle) simultaneously entered as categorical predictors, dummy-coded with reference categories defined a priori (male, intact, Black).

Ordinal logistic regression models were fitted under the proportional odds assumption using the GAMLj module in Jamovi (version 2.6.44.0). This modelling approach assumes parallel slopes across response thresholds. The proportional odds assumption was evaluated using a parallel-lines test (nominal_test function, ordinal package in R, version 4.5.3). Among the models examined, only one statistically significant deviation from proportionality was observed, and it did not involve predictors that were statistically significant in the corresponding proportional odds models.

Predictor effects were evaluated using likelihood ratio chi-square tests. Model fit was assessed using the Akaike Information Criterion (AIC) and McFadden’s pseudo-R^2^. Results are presented as odds ratios (ORs) with 95% confidence intervals (CIs). Statistical significance was set at *p* < 0.05, while *p*-values between 0.05 and 0.10 were interpreted as suggestive trends.

Given the exploratory nature of the study and the large number of item-level tests performed, no formal correction for multiple comparisons was applied, and results should be interpreted cautiously.

Internal consistency of each C-BARQ subscale was assessed using Cronbach’s alpha coefficient, computed through the Reliability Analysis module. All 215 dogs were included in each item-level analysis; sample size did not vary across questionnaire items.

## 3. Results

### 3.1. The Subjects

A total of 276 questionnaires were initially collected. After applying the inclusion criteria (dogs aged between 1 and 8.5 years, clinically healthy, and living with the current owner for at least four months), 61 questionnaires were excluded. The final sample consisted of 215 Australian Shepherd dogs with mean ± SD age of 3.80 ± 2.05 years; 135 dogs lived in a house either without other conspecifics or together with other animals of a different species. The demographic characteristics of the study population are summarized in Table 1.

### 3.2. Survey Results

The behavioral assessment included 15 C-BARQ subscales spanning aggression, fear, excitability, and attachment-related domains. Descriptive statistics and internal consistency coefficients are reported in Table 2.

Australian Shepherds showed high trainability and generally low aggression toward both owners and unfamiliar people, with mean aggression-related scores below 1.5 on a 0–4 scale. Internal consistency was acceptable to high for most subscales (Cronbach’s α = 0.63–0.90), whereas Trainability (α = 0.58) and Chasing (α = 0.40) showed lower reliability.

All statistically significant (*p* < 0.05) and trend-level (*p* < 0.10, flagged with † in tables) regression findings are summarized in Table 3, Table 4 and Table 5, while full item-level results are provided in the Supplementary Material (Tables S1–S15). The proportional odds assumption was generally supported. A single significant deviation from proportionality was detected for coat color in one item, whereas no violations were observed for predictors that were statistically significant in the corresponding proportional odds models. Given the large number of item-level analyses performed, findings based on *p*-values close to the significance threshold should be interpreted with caution.

Most item-level analyses did not reveal significant associations with the predictors examined.

Sex (Table 3) showed statistically significant associations with the largest number of items across behavioral domains.

Females showed lower odds of high-intensity responses in Excitability and Attachment/attention-seeking items. In Dog-directed aggression, sex effects varied by context, with lower odds of aggression toward unfamiliar male dogs and overtly agonistic dogs, but higher odds toward unfamiliar female dogs. Females also showed higher odds of fear responses in selected non-social contexts.

Neuter status (Table 4) showed statistically significant associations with three items. Neutered dogs showed higher odds of aggression toward strangers approaching household members and higher odds of fear responses to thunderstorms and fireworks, while showing lower odds of restlessness when left alone. No other consistent associations were detected.

No statistically significant associations with coat color (Table 5) were observed at the subscale. Significant findings were limited to specific items involving merle-coated dogs. Red Merle dogs showed higher odds of fear-related responses in unfamiliar or aversive situations and during veterinary examination. Blue Merle dogs showed higher odds of howling when left alone and a trend-level association with recall off leash. No additional color-related effects were identified.

## 4. Discussion

Throughout the domestication process and subsequent selective breeding for specific functions or companionship, humans have consistently paid attention to coat color, not only for aesthetic reasons but also because of the long-standing belief that coloration may be associated with behavioral traits.

Early experimental work by Belyaev and colleagues [37] on silver foxes demonstrated that selection for reduced fear and aggression was accompanied by correlated morphological changes, including depigmentation, providing foundational evidence for what is now referred to as the “domestication syndrome” [14].

While previous studies have reported associations between coat color and behavior in dogs of other breeds or in multi-breed populations, evidence within individual breeds remains limited [26,27,31,38].

Previous investigations in dogs have reported associations between coat color and behavioral traits in both multi-breed populations and specific breeds, although findings have been inconsistent. For example, coat color has been linked to behavioral variation in Labrador Retrievers using C-BARQ data, and associations between coat color and aggression-related behaviors have been described in English Cocker Spaniels [39]. However, large-scale C-BARQ-based studies have generally emphasized stronger effects of breed, sex, age, and environmental factors than of coat color per se [40,41]. These mixed findings suggest that coat color effects, when present, are likely to be modest and context-dependent rather than reflecting stable temperament dimensions. In this context, the present study provides new, albeit preliminary, single-breed data in Australian Shepherds.

The analysis of data from 215 dogs contributed to a preliminary characterization of breed-typical behavioral traits. However, significant coat color associations were identified in only a limited number of items and did not extend to consistent patterns across subscales, suggesting that coat color effects, if present, are modest and context-dependent. Variables such as pedigree relatedness and socialization history were not available and may have contributed to the observed variation.

Given the exploratory design of the study and the large number of item-level statistical tests performed, the possibility that some statistically significant findings represent false-positive results (Type I error) must be explicitly acknowledged. No formal correction for multiple comparisons (e.g., Bonferroni or false discovery rate adjustments) was applied, as the primary aim was hypothesis generation rather than confirmatory testing. Although the C-BARQ items are organized into 15 validated behavioral subscales, analyses were conducted at the level of individual items to allow a more fine-grained evaluation of behavioral variation within specific contexts, particularly for stimulus-dependent behaviors such as reactions to traffic, veterinary examination, or unfamiliar objects, which may not be adequately captured by aggregated scores. While this approach offers contextual specificity, it substantially increases the number of simultaneous statistical tests, thereby raising the probability of false-positive findings. Consequently, individual significant associations should be interpreted with caution, particularly when not supported by coherent patterns across related behavioral contexts, and future confirmatory studies with appropriate correction procedures will be necessary to determine the robustness of these findings.

The internal consistency of the C-BARQ subscales, assessed using Cronbach’s alpha, was generally acceptable to high across most behavioral domains, supporting the reliability of the questionnaire in this sample. Lower internal consistency in some subscales likely reflects the context-specific and heterogeneous nature of the behaviors assessed rather than measurement unreliability.

Overall, the findings indicate that the analyzed biological factors exert behavior-specific and context-dependent effects, rather than a generalized influence across all behavioral domains, in line with previous research on canine behavior [5,12,42].

Associations between coat color and behavior were observed in a limited number of contexts where dogs with merle coat patterns (particularly Red Merle individuals) showed higher odds of being classified in higher response categories, while Blue Merle dogs showed increased odds of howling and, in some cases, borderline associations with attention-related behaviors.

Importantly, coat color was not associated with any subscale-level behavioral domains or global temperament traits, and the observed associations were restricted to specific, context-dependent item-level responses. These findings therefore do not support the presence of a generalized behavioral profile linked to coat color.

Although speculative, these findings may be tentatively discussed in light of previously hypothesized links between pigmentation-related genes and neural pathways involved in sensory processing or emotional reactivity [17,18]. Given the observational and exploratory nature of the study, causal interpretations should be avoided.

In addition to potential links between pigmentation-related genes and neural pathways, it is important to consider that the merle genotype has been associated with auditory and visual abnormalities in some dogs. The merle phenotype results from a retrotransposon insertion in the SILV gene (also known as PMEL), which affects melanosome development and pigment distribution [43,44]. Allele length variation within this insertion has been associated with variable expressivity of the merle phenotype [43,45]; in homozygous or certain compound genotypes the merle allele has been linked to congenital sensorineural deafness [46,47] and ophthalmologic abnormalities, including microphthalmia and other developmental defects, particularly in the Australian Shepherd breed [48,49,50]. Altered sensory processing associated with such conditions could plausibly influence behavioral responses to environmental stimuli, including unfamiliar situations, moving objects, or veterinary examination. Therefore, the observed associations in the present study may reflect differences in sensory perception or stimulus processing rather than generalized temperament traits. Because no clinical auditory or ophthalmologic assessments were performed, this possibility cannot be evaluated and warrants further investigation in studies integrating genetic and sensory phenotyping.

Sex emerged as the most consistent predictor across multiple behavioral domains, particularly those related to social interaction, attachment, and excitability. Female dogs showed lower odds of high-intensity responses in several owner-directed behaviors, including greeting family members after brief absences, play with household members, following owners around the house, strong attachment to a specific family member, and agitation when owners displayed affection toward others. This pattern could suggest a reduced expression of intense affiliative or attention-seeking behaviors in females compared with males, consistent with earlier studies reporting sex-related differences in attachment [51] and excitability-related traits [52,53,54,55].

In contrast, sex effects were clearly stimulus-dependent in dog–dog and environmental contexts. Females exhibited lower reactivity toward unfamiliar male dogs and dog-directed threatening stimuli, but higher reactivity toward unfamiliar female dogs [56], heavy traffic, and unfamiliar objects. Such bidirectional effects emphasize the importance of considering social context and stimulus characteristics when interpreting sex differences in canine behavior.

It should also be noted that neuter status was not evenly distributed between males and females in the present sample. Although sex and neuter status were included simultaneously in the regression models, thereby statistically adjusting for their mutual effects, residual confounding cannot be entirely excluded. Consequently, sex-related differences should be interpreted with caution, as some effects may be partially influenced by reproductive status.

Similar context-specific sex effects have been described in previous C-BARQ-based [56,57] and observational studies, suggesting that sex-related behavioral differences are not uniform but vary according to the behavioral domain and environmental challenge [8,9,58].

Neuter status showed selective associations with specific behaviors, particularly those related to fear, arousal, and reactivity. Neutered dogs displayed higher odds of intense responses to strangers approaching household members and to noise-related stimuli such as thunderstorms and fireworks, whereas lower odds were observed for restlessness and agitation. These mixed findings suggest that neutering does not exert a uniform behavioral effect but may interact differently with underlying emotional or motivational systems depending on the context. Because of the cross-sectional nature of the study, it is not possible to determine whether the observed behavioral differences preceded neutering or emerged subsequently.

Importantly, neuter status was not significantly associated with most social and attachment-related behaviors, indicating that its behavioral impact may be more pronounced in fear- and arousal-related domains rather than affiliative ones. This pattern aligns with the previous literature reporting [59,60,61] inconsistent or domain-specific effects of neutering on canine behavior and highlights the need for cautious interpretation of neuter-related behavioral changes in clinical and applied settings [61,62,63].

Taken together, the results support the view that sex shows the most consistent associations, neuter status exerts selective effects, and coat color is linked to specific context-dependent behavioral responses rather than validated subscale-level temperament dimensions.

The absence of universal effects across all behaviors reinforces the importance of context-specific analyses and cautions against overgeneralization.

From a veterinary and applied perspective, these findings may be relevant for behavioral assessment, owner counseling, and management strategies, particularly in contexts involving high arousal, novelty, or social challenges. However, the modest effect sizes and relatively low explained variance observed across models indicate that environmental, experiential, and owner-related factors likely play a substantial role in shaping canine behavior.

Taken together, the present findings suggest that biological variables exert context-dependent and domain-specific influences on canine behavior rather than uniform effects across all behavioral domains. Sex showed the most pervasive associations, followed by selective effects of neuter status predominantly in fear- and arousal-related domains. Coat color showed no evidence of broad temperamental effects; the few suggestive item-level associations in merle-coated dogs may reflect sensory differences associated with the merle genotype rather than temperament per se. This pattern of results is consistent with a view of canine behavioral variation as shaped by the interaction of multiple biological and environmental factors, none of which acts as a global determinant of behavioral phenotype.

This study has several limitations. Behavioral data were collected through owner-reported questionnaires and are therefore subject to perception and reporting bias. Recruitment through social media platforms, breed-specific networks, and breeder contacts may have introduced selection bias, as participation was voluntary and likely attracted highly engaged and motivated owners. Such owners may be more attentive to their dogs’ behavior, more involved in training and management, and potentially more likely to own well-socialized individuals. Consequently, the sample may underrepresent dogs with more severe behavioral problems, limiting the generalizability of the findings to the broader Australian Shepherd population. These factors may have contributed to the generally low levels of aggression and fear observed and limit the generalizability of the findings.

Some coat color groups were numerically smaller, potentially limiting statistical power for detecting subtle effects. In addition, the uneven distribution of sexes and neuter status represents a potential source of residual confounding. More balanced sampling or longitudinal studies would be required to disentangle these influences more clearly.

Information on age at neutering, training intensity, owner and handler experience, and environmental variables was not available and could not be included in the analyses. These factors are known to influence canine behavior and may partially confound the observed associations. In particular, owner and handler experience may be especially relevant for the interpretation of trainability-related scores. Future studies should explicitly account for these variables using longitudinal designs.

Kinship among subjects was not formally assessed; although 80% of dogs had a registered pedigree, no genealogical data were available to evaluate potential relatedness between individuals. Behavioral differences may therefore partially reflect shared breeding lines rather than coat color per se.

Additionally, information on housing conditions was not collected, which represents a further limitation given the potential influence of the living environment on behavioral responses.

Overall, the modest effect sizes and the context-dependent nature of the associations observed reinforce the view that canine behavior arises from complex interactions between biological predispositions and environmental, experiential, and owner-related factors.

## 5. Conclusions

The present study provides a preliminary, exploratory characterization of the behavioral profile of Australian Shepherd dogs. Given the observational design, the reliance on owner-reported questionnaire data, the absence of formal correction for multiple comparisons, and the limited sample size for some coat color groups, all findings should be interpreted with caution and regarded as hypothesis-generating rather than confirmatory.

Sex emerged as the most consistent predictor of behavioral variation, followed by selective effects of neuter status on fear- and arousal-related behaviors. Coat color was not associated with broad behavioral tendencies; exploratory item-level analyses identified only a limited number of suggestive associations in merle-coated dogs, which may reflect sensory rather than temperament differences.

Future studies with larger, coat-color-stratified samples, longitudinal designs, clinical sensory assessment, and objective behavioral measures are needed to determine whether these preliminary associations are reproducible and to clarify the underlying biological mechanisms.

## Figures and Tables

**Table 1:** Study population characteristics.

Characteristic	Category	*n*	%
Sex	Male	104	48.37
	Female	111	51.63
Neuter status	Male—intact	91	87.50
	Male—neutered	13	12.50
	Female—intact	51	45.95
	Female—spayed	60	54.05
Coat color	Blue Merle	75	34.88
	Black	62	28.84
	Red	40	18.60
	Red Merle	38	17.67
Age (years)	Mean ± SD	3.80 ± 2.05	—
	Range	1.0–8.5	—
Age at adoption (months)	Mean ± SD	4.45 ± 8.05	—
Living arrangements	Without conspecifics or with other species only	135	62.79
	With at least one conspecific	80	37.21
Total		215	100

**Table 2:** C-BARQ subscale scores (descriptive statistics).

#	Subscale	Mean ± SD	Median	IQR	Cronbach α
1	Trainability ^a^	3.35 ± 0.41	3.40	3.20–3.60	0.58
2	Stranger-directed aggression ^b^	0.73 ± 0.68	0.60	0.20–1.10	0.90
3	Owner-directed aggression ^b^	0.13 ± 0.34	0.00	0.00–0.00	0.81
4	Dog rivalry ^b^	0.50 ± 0.67	0.25	0.00–0.75	0.78
5	Stranger-directed fear ^b^	0.74 ± 0.79	0.50	0.25–1.00	0.90
6	Non-social fear ^b^	0.86 ± 0.66	0.71	0.29–1.29	0.70
7	Dog-directed fear ^b^	0.66 ± 0.66	0.50	0.25–1.00	0.87
8	Touch sensitivity ^b^	0.30 ± 0.55	0.00	0.00–0.33	0.63
9	Dog-directed aggression ^b^	1.10 ± 0.75	1.00	0.50–1.50	0.83
10	Separation-related behavior ^a^	1.45 ± 0.48	1.25	1.12–1.62	0.73
11	Excitability ^a^	2.31 ± 0.93	2.25	1.67–3.00	0.84
12	Attachment and attention-seeking ^a^	3.55 ± 0.82	3.64	3.00–4.14	0.75
13	Chasing ^a^	1.27 ± 1.19	1.00	0.50–2.00	0.40
14	Energy level ^a^	1.90 ± 1.03	1.67	1.33–2.83	0.72
15	Miscellaneous ^c^	0.61 ± 0.38	0.57	0.43–0.88	0.74

ᵃ Scored on a 0–5 scale (frequency). ᵇ Scored on a 0–4 scale (intensity). ᶜ Mixed scale (both frequency and intensity items). Higher scores indicate more problematic behaviors, except for certain Trainability items where higher scores reflect more desirable traits.

**Table 3:** Regression results for sex.

Sex				
Subscale	Item and Direction of Effect (→)	OR	95% CI	χ^2^ (df); *p*
Non-social fear	Q36. The dog acts anxious or fearful in heavy traffic *→ Females show higher fear intensity than males*	2.07	1.14–3.80	χ^2^ = 5.77 (1); *p* = 0.016
	Q37. The dog acts anxious or fearful in response to strange and unfamiliar objects on or near sidewalks (e.g., garbage bags, leaves, litter, waving flags) *→ Females show higher fear intensity than males*	2.13	1.16–3.97	χ^2^ = 6.01 (1); *p* = 0.014
Dog-directed aggression	Q49. The dog acts aggressively when approached directly by an unknown male dog while out walking on a leash *→ Females show lower aggression intensity than males*	0.27	0.15–0.47	χ^2^ = 21.70 (1); *p* < 0.001
	Q50. The dog acts aggressively when approached directly by an unknown female dog while out walking on a leash *→ Females show higher aggression intensity than males*	2.07	1.18–3.67	χ^2^ = 6.41 (1); *p* = 0.011
	Q52. The dog acts aggressively when an unknown dog barks, growls or lunges at him *→ Females show lower aggression intensity than males*	0.51	0.30–0.88	χ^2^ = 5.94 (1); *p* = 0.015
Excitability	Q61. The dog is excited when a household member returns home after a short period of absence *→ Females show lower excitability than males*	0.50	0.29–0.87	χ^2^ = 6.19 (1); *p* = 0.013
	Q62. The dog is excited when playing with household members *→ Females show lower excitability than males*	0.50	0.29–0.85	χ^2^ = 6.44 (1); *p* = 0.011
	Q63. The dog is excited when the intercom/bell rings *→ Females show lower excitability than males*	0.56	0.33–0.97	χ^2^ = 4.35 (1); *p* = 0.037
	Q65. The dog is excited just before being driven around in the car *→ Females show lower excitability than males*	0.50	0.29–0.85	χ^2^ = 6.45 (1); *p* = 0.011
Attachment/attention-seeking	Q67. The dog shows strong attachment to a specific family member *→ Females show lower attachment frequency than males*	0.51	0.29–0.90	χ^2^ = 5.34 (1); *p* = 0.021
	Q68. The dog tends to follow household members around the house, from room to room *→ Females show lower following frequency than males*	0.44	0.25–0.78	χ^2^ = 7.99 (1); *p* = 0.005
	Q71. The dog gets upset if owners show affection for another person *→ Females show lower frequency of this behavior than males*	0.50	0.29–0.87	χ^2^ = 6.12 (1); *p* = 0.013
	Q72. The dog gets agitated when owners show affection for another dog/animal *→ Females show lower frequency of this behavior than males*	0.55	0.32–0.93	χ^2^ = 4.91 (1); *p* = 0.027

Note. Only items with *p* < 0.05 are reported. OR = odds ratio; CI = confidence interval. Reference categories: male sex.

**Table 4:** Regression results for neuter status.

Neuter Status				
Subscale	Item and Direction of Effect (→)	OR	95% CI	χ^2^ (df); *p*
Stranger-directed aggression	Q12. The dog acts aggressively when a stranger approaches the owner or another household member while at home *→ Neutered dogs show higher aggression intensity than intact dogs*	2.23	1.18–4.28	χ^2^ = 6.13 (1); *p* = 0.014
Non-social fear	Q38. The dog acts anxious or fearful during thunderstorms, fireworks or similar events *→ Neutered dogs show higher fear intensity than intact dogs*	1.90	1.06–3.42	χ^2^ = 4.62 (1); *p* = 0.032
Separation-related behavior	Q55. The dog shows restlessness/agitation when left or about to be left alone *→ Neutered dogs show lower restlessness frequency than intact dogs*	0.45	0.23–0.88	χ^2^ = 5.55 (1); *p* = 0.019

Note. Only items with *p* < 0.05 are reported. OR = odds ratio; CI = confidence interval. Reference category: intact status.

**Table 5:** Regression results for coat color.

Coat Color				
Subscale	Item and Direction of Effect (→)	OR	95% CI	χ^2^ (df); *p*
Trainability	Q1. The dog, when off leash, returns immediately when called *→ Blue Merle dogs show higher recall frequency than Black dogs*	1.95	1.00–3.81	χ^2^ = 6.40 (3); *p* = 0.050
Non-social fear	Q37. The dog acts anxious or fearful in response to strange and unfamiliar objects on or near sidewalks *→ Red Merle dogs show higher fear intensity than Black dogs*	2.06	0.95–4.50	χ^2^ = 5.80 (3); *p* = 0.084 †
	Q39. The dog acts anxious or fearful when in an unfamiliar situation for the first time (e.g., first car trip, first visit to the vet) *→ Red Merle dogs show higher fear intensity than Black dogs*	2.31	1.10–4.89	χ^2^ = 10.87 (3); *p* = 0.027
	Q40. The dog acts anxious or fearful in response to wind or objects moved by the wind *→ Red Merle dogs show higher fear intensity than Black dogs*	3.10	1.40–6.93	χ^2^ = 12.78 (3); *p* = 0.005
Touch sensitivity	Q45. The dog acts anxious, fearful or aggressive when visited/treated by vet *→ Red Merle dogs show higher touch sensitivity than Black dogs*	2.47	1.18–5.24	χ^2^ = 13.66 (3); *p* = 0.017
Separation-related behavior	Q58. The dog howls when left or about to be left alone *→ Blue Merle dogs show higher howling frequency than Black dogs*	2.99	1.21–8.20	χ^2^ = 9.26 (3); *p* = 0.026

Note. Only items with *p* < 0.05 or suggestive trend *p* < 0.10 (†) are reported. OR = odds ratio; CI = confidence interval. Reference category: black color.

## Data Availability

The original contributions presented in this study are included in the article/supplementary material. Further inquiries can be directed to the corresponding author.

## Supplementary Materials

The following supporting information can be downloaded at: https://www.mdpi.com/article/10.3390/vetsci13030299/s1. Tables S1–S15: Item-level descriptive and inferential results for each of the 15 C-BARQ behavioral subscales (S1:Trainability, S2;Stranger-Directed Aggression, S3: Owner-Directed Aggression, S4: Dog Rivalry, S5: Stranger-Directed Fear, S6: Non-Social Fear, S7: Dog-Directed Fear, S8:Touch Sensitivity, S9: Dog-Directed Aggression, S10: Separation-Related Behavior, S11: Excitability, S12; Attachment/Attention-Seeking, S13: Chasing, S14: Energy Level, S15:Miscellaneous).
