# Supplementary material for: Behavior in Australian Shepherd Dogs Assessed Using the C-BARQ: A Preliminary Study of Associations with Coat Color, Sex, and Neutering Status
Source: Vet Sci. 2026 Mar 21;13(3):299. doi: 10.3390/vetsci13030299 (PMC13030206; doi:10.3390/vetsci13030299)
Supplement: Supplementary file 1 [file vetsci-13-00299-s001.zip › vetsci-4193870-supplementary.pdf]

Table S1. Item-level results for the **Trainability** subscale. For each item, the mean score (± SD) is reported. Statistically significant and trend-level associations with sex, neuter status, and coat color are reported as odds ratios (ORs) with 95% confidence intervals (CIs) and p values derived from ordinal logistic regression models. Reference categories were male sex, intact reproductive status, and black coat color.

Question<br>The dog:	Mean ± SD
1. When off leash, returns immediately when called	4.25 ± 1.01
2. Immediately obeys the 'sit' command	4.40 ± 0.74
3. Immediately obeys the 'stay' command	3.74 ± 1.24
4. Appears to pay attention to/listen carefully to everything you say and/or do	4.45 ± 0.77
5. Does not respond promptly to corrections or punishments, has a 'thick skin'	2.62 ± 1.27
6. He is slow in learning new exercises/tasks	1.85 ± 0.86
7. It is easily distracted by noises, smells or the sight of interesting things	3.33 ± 1.10
8. Bring back or try to bring back sticks, balls or other objects	3.71 ± 1.34

Table S2. Item-level results for the **Stranger-Directed Aggression** subscale. See Table S1 for reporting conventions.

STRANGER DIRECT AGGRESSION<br>Intensity scale (0-4)	
Question<br>The dog acts aggressively:	Mean ± SD
9. When approached directly by an unknown adult while out for a walk on a leash	0.61 ± 0.96
10. When approached directly by an unknown child, while walking on a leash	0.75 ± 1.14
11. Towards unknown people who approach the dog when he is in the car (for example, while he is refueling)	0.61 ± 1.05
12. When a stranger approaches you or another household member while you are at home	0.71 ± 1.01
13. When strangers approach you or another family member while you are away from home	0.60 ± 0.87
14. When a deliveryman or postman approaches your home	1.28 ± 1.26
15. When strangers pass by the house while the dog is in the garden/yard	1.35 ± 1.20
16. When an unknown person tries to touch or pet the dog	0.62 ± 1.01
17. When cyclists, joggers, people on skateboards or rollerblades pass the house while the dog is in the yard/garden	1.13 ± 1.24
18. Towards unknown people who come to visit your home	0.71 ± 1.06

Table S3. Item-level descriptive statistics for the **Owner-Directed Aggression** subscale. Mean (± SD) scores are reported on an intensity scale (0–4). No statistically significant associations with sex, neuter status, or coat color were detected.

OWNER-DIRECTED AGGRESSION<br>Intensity scale (0-4)	
Question<br>Dog acts aggressively:	Mean ± SD
19. When verbally corrected or punished (reprimanded, scolded, etc.) by you or another family member	0.13 ± 0.42
20. When playing, bones or other objects are taken away by a member of the household	0.18 ± 0.54
21. When washed or brushed by a member of the household	0.14 ± 0.50
22. When approached directly by you or another member of the household, while (the dog) is eating	0.12 ± 0.47
23. When food is taken away by a family member	0.14 ± 0.54
24. When looked straight in the eye by a family member	0.11 ± 0.45
25. When a family member steps over him, he passes over him	0.13 ± 0.55
26. When you or another household member takes back food or items previously stolen by the dog	0.16 ± 0.58

Table S4. Item-level descriptive and inferential results for the **Dog Rivalry** subscale.

DOG RIVALRY<br>Intensity scale (0-4)	
Question<br>Dog acts aggressively:	Mean ± SD
27. Towards another dog living in the same house (leave blank if there are no other dogs in the house)	0.38 ± 0.73
28. When approached by another dog living in the same house while he is in his favorite kennel/resting place (leave blank if there are no other dogs in the house)	0.33 ± 0.75
29. When approached by another dog living in the same household while eating (leave blank if there are no other dogs in the house)	0.88 ± 1.21

30. When approached by another dog living in the same household while chewing/playing with a favorite toy, bone, object etc. (leave blank if there are no other dogs in the house)	0.75 ± 0.98

Table S5. Item-level descriptive and inferential results for the **Stranger-Directed Fear** subscale.

STRANGER-DIRECTED FEAR<br>Intensity scale (0-4)	
Question<br>Dog acts anxious or fearful:	Mean ± SD
31. When approached directly by an unknown adult person, not at home	0.66 ± 1.00
32. When approached directly by an unknown child, not at home	0.72 ± 1.05
33. When unknown people come to your house	0.50 ± 0.97
34. When a stranger tries to touch or pet the dog	0.60 ± 0.96

Table S6. Item-level descriptive and inferential results for the **Non-Social Fear** subscale.

NON-SOCIAL FEAR<br>Intensity scale (0-4)	
Question<br>Dog acts anxious or fearful:	Mean ± SD
35. In response to sudden or loud noises (e.g: vacuum cleaners, car engines, street work, accidentally dropped objects)	1.16 ± 1.22
36. In heavy traffic	0.69 ± 0.93
37. In response to strange and unfamiliar objects on or near sidewalks (e.g. garbage bags, leaves, litter, waving flags, etc.)	0.62 ± 0.89
38. During thunderstorms, fireworks or similar events	1.52 ± 1.59
39. When you find yourself, for the first time, in unfamiliar situations (e.g.: the first car trip, the first time in the elevator, the first visit to the vet)	0.83 ± 0.99
40. In response to wind or objects moved by the wind	0.64 ± 0.97

Table S7. Item-level descriptive and inferential results for the **Dog-Directed Fear** subscale

DOG-DIRECTED FEAR<br>Intensity scale (0-4)	
Question<br>Dog acts anxious or fearful:	Mean ± SD
41. When approached directly by an unknown dog of the same size or larger	0.93 ± 1.08
42. When approached directly by a smaller dog	0.50 ± 0.81
43. When unknown dogs come to your house	0.53 ± 0.92
44. When an unknown dog barks, growls or lunges at him	1.08 ± 1.14

Table S8. Item-level descriptive and inferential results for the **Touch Sensitivity** subscale.

TOUCH SENSITIVITY<br>Intensity scale (0-4)	
Question<br>Dog acts anxious or fearful or aggressive:	Mean ± SD
45. When visited/treated by vet	1.21 ± 1.21
46. When their nails are cut by a family member	0.42 ± 0.79
47. When brushed or washed by a member of the household	0.35 ± 0.69
48. When his paws are dried by a member of the household	0.25 ± 0.63

Table S9. Item-level descriptive and inferential results for the **Dog-Directed Aggression** subscale.

DOG-DIRECTED AGGRESSION<br>Intensity scale (0-4)	
Question<br>Dog acts aggressively:	Mean ± SD
49. When approached directly by an unknown male dog while out walking on a leash	1.33 ± 1.35
50. When approached directly by an unknown female dog while out walking on a leash	0.90 ± 1.12

51. Towards unknown dogs visiting your home	1.00 ± 1.18
52. When an unknown dog barks, growls or lunges at him	2.18 ± 1.43

Table S10. Item-level descriptive and inferential results for the **Separation-Related Behavior** subscale.

SEPARATION-RELATED BEHAVIOR<br>Frequency scale (0-5)	
Question<br>The dog when left or about to be left on its own:	Mean ± SD
53. Shakes, shivers, trembles	1.28 ± 0.74
54. Has excessive salivation	1.26 ± 0.64
55. Shows restlessness agitation walking incessantly	1.68 ± 1.04
56. Whimpers	1.73 ± 1.05
57. Barks	1.95 ± 1.09
58. Howls	1.33 ± 0.77
59. Shows chewing scratching doors, windows, floors, curtains, etc.	1.45 ± 0.85
60. Has loss of appetite	1.27 ± 0.70

Table S11. Item-level descriptive and inferential results for the **Excitability** subscale.

EXCITABILITY<br>Intensity scale (0-4)	
Question<br>The dog is excited:	Mean ± SD
61. When you or another household member returns home after a short period of absence	2.80 ± 1.06
62. When he plays with you or other family members	2.62 ± 1.05
63. When the intercom/bell rings	2.50 ± 1.31
64. Just before being taken out for a walk	2.19 ± 1.37
65. Just before being driven around in the car	1.67 ± 1.45

66. When people come home	2.40 ± 1.23

Table S12. Item-level descriptive and inferential results for the **Attachment/Attention-Seeking** subscale.

ATTACHMENT/ATTENTION-SEEKING<br>Frequency scale (0-5)	
Question<br>The dog is:	Mean ± SD
67. Shows a strong attachment to a specific family member	3.98 ± 1.20
68. Tends to follow you (or other household members) around the house, from room to room	4.16 ± 1.06
69. Tends to sit close to or touching you (or others) when you are sitting	4.14 ± 0.86
70. Tends to nudge or paw to get attention from you (or others) when you are sitting	3.45 ± 1.24
71. Gets upset (whimpers, jumps at you, tries to get in the way) if you (or others) show affection for another person	3.25 ± 1.48
72. Gets agitated (whispers, jumps on you, tries to get in the way) when you (or others) show affection for another dog/animal	3.26 ± 1.38

Table S13. Item-level descriptive and inferential results for the **Chasing** subscale.

CHASING<br>Frequency scale (0-5)	
Question<br>The dog:	Mean ± SD
73. Acts aggressively toward cats, squirrels, and other animals entering its yard	1.46 ± 1.31
74. Chases or would chase cats given the opportunity	2.54 ± 1.67
75. Chases or would chase birds given the opportunity	2.03 ± 1.18

76. Chases or would chase squirrels, rabbits and other small animals given the opportunity	2.23 ± 1.82

Table S14. Item-level descriptive and inferential results for the **Energy Level** subscale.

ENERGY LEVEL<br>Frequency scale (0-5)	
Question<br>The dog:	Mean ± SD
77. Is playful, puppyish, and boisterous	3.57 ± 1.27
78. Is active, energetic, and always on the go	2.94 ± 1.26

Table S15. Item-level descriptive and inferential results for the **Miscellaneous** subscale.

MISCELLANEOUS<br>Frequency scale (0-5)	
Question<br>The dog:	Mean ± SD
79. Escapes or would escape home or yard given a chance	1.17 ± 0.75
80. Rolls in animal droppings or other 'smelly' substances	1.80 ± 1.35
81. Eats own or other animals' droppings or feces	1.57 ± 1.12
82. Chews inappropriate objects	1.71 ± 1.07
83. Mounts objects, furniture, or people	1.40 ± 0.88
84. Begs persistently for food when people are eating	3.20 ± 1.41
85. Steals food	2.11 ± 1.25
86. Nervous or frightened on stairs	1.60 ± 1.17
87. Pulls excessively hard when on the leash	2.74 ± 1.17
88. Urinates against objects/furnishings in your home	0.89 ± 0.32
89. Urinates when approaches, petted, handled or picked up	1.11 ± 0.63
90. Urinates when left alone at night, or during the daytime	1.00 ± 0.34
91. Defecates when left alone at night, or during the daytime	0.94 ± 0.24

92. Hyperactive, restless, has trouble settling down	2.17 ± 1.04
93. Stares intently at nothing visible	1.40 ± 0.95
94. Snaps at (invisible) flies	1.34 ± 0.73
95. Chases own tail/hind end	1.23 ± 0.81
96. Chases/follows shadows, light spots, etc.	0.74 ± 0.44
97. Barks persistently when alarmed or excited	2.60 ± 1.35
98. Licks him/herself excessively	1.86 ± 1.09
99. Licks people or objects excessively	2.11 1.28
100. Displays other bizarre, stranger or repetitive behavior(s)	1.34 ± 1.06
